# Regular Inhaled Corticosteroids Use May Protect Against Severe COVID-19 Outcome in COPD

**DOI:** 10.2147/COPD.S404913

**Published:** 2023-08-07

**Authors:** Marina Labor, Brian K Kirui, Fredrik Nyberg, Lowie E G W Vanfleteren

**Affiliations:** 1 Cancer and Lung Health Care Unit, University Hospital in Linköping, Linköping, Sweden; 2 School of Public Health and Community Medicine, Institute of Medicine, Sahlgrenska Academy, University of Gothenburg, Gothenburg, Sweden; 3 COPD Center, Department of Respiratory Medicine and Allergology, Sahlgrenska University Hospital, Gothenburg, Sweden; 4 Department of Internal Medicine and Clinical Nutrition, Institute of Medicine, Sahlgrenska Academy, University of Gothenburg, Gothenburg, Sweden

**Keywords:** COVID −19, COPD, mortality

## Abstract

**Purpose:**

Population-based studies provide conflicting evidence about how inhaled corticosteroids (ICS) impact COVID-19 outcomes among COPD patients. We investigated whether regular ICS exposure affects risk, severity, or survival in SARS-CoV-2 infection, using a nationwide linked Swedish population register database.

**Patients and Methods:**

During January–December 2020, we studied two defined Swedish adult populations – Whole population [≥40 years] (N = 5243479), and COPD subpopulation [≥40 years] (N = 133372), in three study cohorts, respectively: 1. Overall cohort (index date 1 Jan 2020), 2. COVID-19 diagnosed sub-cohort (index date = diagnosis date), and 3. COVID-19 hospitalized sub-cohort (index date = admission date). Regular exposure was defined as ≥3 ICS prescriptions in the year before index. Hazard ratios (HRs) for outcomes (COVID-19 onset, hospitalization, ICU admission, or death) related to ICS exposure were estimated using Cox regression. Confounding was controlled by propensity score methods applying Average Treatment effect in the Treated (ATT) weighting.

**Results:**

Regular ICS use was associated with only very slightly increased onset of COVID-19, hospitalization, ICU admission, and death in the overall whole population cohort and in the overall COPD subpopulation cohort, except for ICU admission (marginally non-significant HRs, up to 1.13); and no clear increase in the diagnosed sub-cohorts. However, in the COVID-19 hospitalized COPD sub-cohort, ICS therapy showed reduced risks against progression to ICU admission and death, significant for death (HR 0.82 95% CI [0.67–0.99]).

**Conclusion:**

For COPD patients, ICS therapy offers some protection against progression to ICU admission and death among COVID-19 hospitalized patients. Our findings alleviate concerns about increased risks of COVID-19 by ICS treatment and provide evidence supporting the continuation of ICS therapy for COPD patients.

## Introduction

Patients with chronic obstructive pulmonary disease (COPD) are of particular interest in relation to coronavirus disease 2019 (COVID-19) because COPD is a highly prevalent disease that is associated with impaired lung function. Overall, COPD patients seem not to have an increased risk of acquiring severe acute respiratory syndrome coronavirus 2 (SARS-CoV-2) infection.^1^ In contrast, it has been shown that COPD patients with COVID-19 have more than fivefold increased risk of a severe disease course requiring intensive care unit (ICU) support, and higher mortality in comparison to other patient groups,^2^ which is at least partly explained by higher age and comorbidity prevalence.^3–6^

Inhaled corticosteroids (ICS) are commonly used in patients with COPD to prevent exacerbations and/or control symptoms. Early in the COVID-19 pandemic, concerns were raised related to potential impaired antiviral response associated with ICS use, due to suppression of interferon-mediated innate and adaptive immunity.^7^ Consequently, ICS users would have higher risk for severe illness if they acquired COVID-19. To date, published (mostly laboratory) studies and reviews rather suggest a potential preventive effect of pre-morbid and continued use of ICS against a hyperinflammatory response.^8–10^ A recent study, in contrast, reported that pre-morbid ICS use in COPD was not protective and even increased risk of developing COVID-19.^11^ A systematic review of more recent studies about pre-morbid and continued use of ICS found no evidence that ICS use was associated with worse outcomes, even though results and conclusions are likely confounded by different factors (ICS indication, sample size, lack of adjustment for comorbidities).^6^ Current recommendations are that standard maintenance and exacerbation ICS therapy should not be discontinued, because maintenance of optimal pharmacologic treatment generally is the best way to prevent exacerbations and/or reduce the severity of exacerbations, including those caused by viruses.^12^ In people who do not have COPD, ICS prescription initiated in mild COVID-19 appears to reduce the risk of admission to hospital or death and reduce the duration of symptoms.^13^ Besides, both ICS and oral corticosteroids (OCS) have been shown to be effective in the treatment of severe SARS-CoV-2 infection with respiratory failure in the advanced phase by reducing the hyperinflammatory response of the host and beneficially affecting the severity of acute lung injury.^14^,^15^ Due to the conflicting results summarized above, our primary objectives were to investigate, using a large Swedish population linked register database, whether ongoing regular ICS exposure may affect risk of onset, or severity (hospitalization, ICU admission or death) outcomes of SARS-CoV-2 infection in the whole population [≥40 years], as well as to investigate the effect of ICS use on these risks specifically in COPD patients, a vulnerable patient group with frequent use of ICS, within this same population database.

## Materials and Methods

### Study Design, Data Sources and Study Population

We conducted a cohort study in a Swedish nationwide population of individuals aged 40 years and above and free of COVID-19 on 1 Jan 2020 (N = 5243 479). Data come from the SCIFI-PEARL (Swedish COVID-19 Investigation for Future Insights – a Population Epidemiology Approach using Register Linkage) project, described in detail elsewhere.^16^ This project database, now extended to the entire Swedish population, includes all individuals who developed COVID-19 in the Swedish population, identified using a range of register data.

For the current study, we defined two study populations of Swedish adults ≥40 years: 1) whole population, and 2) COPD subpopulation, and within these populations studied three different study cohorts (with corresponding index dates): an overall cohort (index date 1 Jan 2020), a COVID-19 diagnosed sub-cohort (individuals with COVID-19 infection onset with infection date as their index date), and hospitalized sub-cohort (COVID-19 patients with admission date as their index date). The overall cohort and sub-cohorts of COPD patients included individuals with COPD on 1 Jan 2020, identified based on International Classification of Diseases, revision 10 (ICD-10) diagnostic codes J41–J44 related to specialist care visits or hospital admissions in the National Patient Register (NPR) or being registered as COPD patients in the Swedish National Airway Register (SNAR), from 1 Jan 2015 until 31 Dec 2019.

Beyond the two data sources already mentioned, used for defining the study population, baseline comorbidities and some COVID-19 outcomes, other data sources within SCIFI-PEARL used in this study were: 1. SmiNet (the national database of notifiable diseases) for diagnosis based on reporting positive PCR result by doctors and/or laboratories; 2. The Swedish Intensive Care Register (SIR) for admissions to ICU; 3. The Cause-of-Death Register (CDR) for COVID-19 death; 4. The National Prescribed Drug Register (NPDR) for treatments with prescription medications; and 5. National sociodemographic registers, ie, the Register of Total Population (RTB), and Longitudinal Integrated Database for Health Insurance and Labor Market Studies (LISA) from Statistics Sweden with data on immigration, emigration, deaths and sociodemographic characteristics. The linked data thus comprehensively contained detailed information for each subject regarding COVID-19, demographics, comorbidities, prescribed medications, and clinical data for COPD patients. The study has ethical approval from the Swedish Ethical Review Authority and complies with the Declaration of Helsinki. Informed consent was not required, since the study is based on secondary healthcare data.

### Exposure, Outcomes, and Follow-Up

The main study exposure was regular ongoing ICS exposure, defined as the presence of at least three filled ICS prescriptions (with Anatomical Therapeutic Chemical (ATC) codes R03BA, R03AK, R03AL08, and R03AL09) within the year before index date in the NPDR. Non-exposed individuals received less than 3 prescriptions of ICS in the same period. This definition was chosen to capture a patient’s consistent and continuous use of the medication over this time and likely continued use after the index date into the study follow-up period. Swedish prescriptions normally cover approximately 3 months of use, so mostly this would mean continuous use; our data also confirmed that with this definition >85% of individuals in the whole population and COPD population in our study who were exposed to ICS had their last prescriptions filled within 100 days and are likely to be regular users.

We defined COVID-19 outcomes as: COVID-19 onset (individuals with positive test result for SARS-CoV-2 in SmiNet, or an out- or inpatient primary or secondary diagnosis with ICD-10 U07.1 or U07.2 in the NPR; event date was the earliest of these), hospitalization (primary or secondary diagnosis U07.1 or U07.2 from inpatient care in the NPR; event date was the date of admission), intensive care unit (ICU) admission (U07.1 or U07.2 from SIR; event date being date of admission) and death (underlying or contributing cause of death U07.1 or U07.2 in the CDR).

In all analyses, follow-up started on the respective cohort index date, and extended to the earliest of outcome, emigration, death, or end of follow-up on 31 Dec 2020.

### Covariates

We included the following covariates: sociodemographic factors from national sociodemographic registers were age (≥40 years), gender, employment status (unemployed 40–64 years, employed 40–64, employed 65+ and retired), education (primary, secondary, or higher). Comorbidities were defined based on ICD-10 codes registered during 2015–2019 in the NPR, including hypertension, cardiovascular disease, heart failure, stroke, ischemic heart disease (IHD), diabetes, respiratory disease, COPD, asthma, respiratory failure, lung cancer, rheumatologic and immunologic disease, chronic kidney disease and kidney failure. Drug exposures were based on ATC codes in NPDR in 2019, and included oral corticosteroids, biologics, short- and long-acting bronchodilator agonists, short- and long-acting muscarinic antagonists, leukotriene receptor antagonists, immunosuppressants, and statins. Patient factors that were obtained from SNAR registrations during 2015–2019 included body mass index, smoking, and post-bronchodilator Forced Expiratory Volume in one second, percent of predicted (FEV_1_% predicted) based on the Swedish Hedenström reference values.^17^ Missing data were common for patient factors as they were not recorded on every visit and were addressed by 1) using the last observations carried forward 2) missing post-bronchodilator FEV_1_ values replaced with pre-bronchodilator FEV_1_ values 3) supplementing smoking and BMI data from similar information in other registers as described in Table S1. In addition, our analytic strategy included gradient boosting modeling which handles missing as a separate category in the propensity score estimation, thereby balancing the proportions of missing between the exposure groups.^18^

### Statistical Analysis

The study populations were characterized using absolute and relative frequencies for categorical variables, and averages and standard deviations for continuous variables. The number and incidence rates of events were computed.

Hazard ratios (HRs) and 95% confidence intervals (CI) for studied outcomes related to ICS exposure were estimated using Cox regression models. Propensity score analysis using Average Treatment effect in the Treated (ATT) weighting was applied in the Cox regression models to control the effect of confounding when comparing exposed and corresponding non-exposed groups. Propensity scores were estimated using a gradient boosted logistic regression model (twang package in R), including all potential confounders in the model, and optimizing the number of trees to give the best possible balance as measured by the lowest average standardized mean differences (SMD) across the potential confounders.^18^ The balance between the exposed and non-exposed groups was evaluated prior to and after propensity score weighting using the SMD and considered satisfactory when the SMD was <0.25, or preferably <0.1.^19^ If satisfactory balance overall could not be achieved, a “doubly robust” model was estimated, including imbalanced covariates (SMD >0.1) also in the outcome model.^20^ R (version 4.0.2) for statistical computing was used for all analyses.

## Results

In the overall (population-based, pre-COVID-19) cohorts, 3% (186,577/5,243,479) of the whole population and 35.7% (47,559/133,372) of COPD patients were regularly exposed to ICS (Table 1). The mean age of the ICS-exposed tended to be higher than for the non-exposed in the overall cohorts and COVID-19 diagnosed sub-cohorts, especially for the whole population (Table 1, Table S2, respectively), but this difference was less obvious in the COVID-19 hospitalized sub-cohorts (Table S3). ICS-exposed in all cohorts were more often women. The ICS-exposed tended to have more comorbidities and higher medication use than the non-exposed in the whole population cohorts and sub-cohorts, but this was not consistently seen in the COPD cohorts (Table 1, Tables S2 and S3). The low SMDs after propensity score weighting for most covariates indicated that a very good balance was achieved between the exposed and non-exposed after weighting for most cohorts, but less satisfactory for the whole population overall cohort (Table 1, Tables S2 and S3). For this cohort, the doubly robust analysis was thus implemented.

**Table 1:** Demographic Characteristics, Comorbidities, and Prescribed Medications of the Overall Cohorts of the Whole Population and COPD Patients on 1 Jan 2020, for Individuals ≥40 Years of Age Exposed and Non-Exposed to Prior Regular Treatment with Inhaled Corticosteroids (ICS), with Standardized Mean Differences (SMD) to Assess Differences for All Characteristics Between the Cohorts Before (Crude) and After ATT Weighting by Propensity Scores (Weighted)

	Whole Population (N = 5,243,479)				COPD Patients (N = 133,372)			
	Non-Exposed*	Exposed**			Non-Exposed*	Exposed**		
n (%)	5,056,902 (96.4%)	186,577 (3.6%)	SMD (Crude)	SMD (Weighted)	85,813 (64.3%)	47,559 (35.7%)	SMD (Crude)	SMD (Weighted)
**Demographics**								
Age (mean ± SD)	60.8 (13.5)	66.1 (12.9)			72.7 (10.0)	73.4 (9.6)		
Age categories (years)			0.419	0.219			0.075	0.008
40–49	1,282,612 (25.4%)	23,795 (12.8%)			1546 (1.8%)	662 (1.4%)		
50–59	1,265,403 (25.0%)	35,980 (19.3%)			7374 (8.6%)	3502 (7.4%)		
60–69	1,064,972 (21.1%)	44,788 (24.0%)			20,526 (23.9%)	10,629 (22.3%)		
70–79	936,739 (18.5%)	52,789 (28.3%)			34,838 (40.6%)	20,270 (42.6%)		
80–89	412,202 (8.2%)	24,568 (13.2%)			18,613 (21.7%)	10,816 (22.7%)		
90+	94,974 (1.9%)	4657 (2.5%)			2916 (3.4%)	1680 (3.5%)		
Gender = women	2,557,068 (50.6%)	114,079 (61.1%)	0.214	0.080	45,853 (53.4%)	28,437 (59.8%)	0.129	<0.001
Employment			0.389	0.192			0.075	0.005
Unemployed 40–64 years	413,324 (8.2%)	14,960 (8.0%)			7176 (8.4%)	3639 (7.7%)		
Employed 40–64 years	2,679,672 (53.0%)	65,929 (35.3%)			10,101 (11.8%)	4716 (9.9%)		
Employed ≥ 65 years	532,337 (10.5%)	22,594 (12.1%)			10,640 (12.4%)	5614 (11.8%)		
Retired ≥ 65 years	1,428,055 (28.3%)	83,093 (44.5%)			57,892 (67.5%)	33,590 (70.6%)		
Education			0.151	0.053			0.013	0.006
Primary	959,029 (19.3%)	44,999 (24.3%)			30,992 (36.6%)	17,303 (36.8%)		
Secondary	2,201,763 (44.2%)	83,593 (45.2%)			39,167 (46.3%)	21,866 (46.6%)		
Higher	1,816,319 (36.5%)	56,408 (30.5%)			14,465 (17.1%)	7796 (16.6%)		
**Comorbidities**								
Hypertension	723,351 (14.3%)	50,297 (27.0%)	0.317	0.084	38,932 (45.4%)	22,334 (47.0%)	0.032	0.001
Cardiovascular disease	1,096,906 (21.7%)	70,877 (38.0%)	0.362	0.102	52,708 (61.4%)	30,131 (63.4%)	0.040	0.003
Heart failure	124,714 (2.5%)	13,384 (7.2%)	0.221	0.061	13,329 (15.5%)	8255 (17.4%)	0.049	0.001
IHD	237585 (4.7%)	16,815 (9.0%)	0.171	0.029	16,528 (19.3%)	8832 (18.6%)	0.018	<0.001
Stroke	87,504 (1.7%)	4725 (2.5%)	0.056	0.032	4757 (5.5%)	2252 (4.7%)	0.037	0.002
Diabetes	290,310 (5.7%)	17,849 (9.6%)	0.144	0.017	14,429 (16.8%)	7411 (15.6%)	0.033	0.004
Respiratory disease	230,319 (4.6%)	81,305 (43.6%)	1.026	0.277	59,369 (69.2%)	39,095 (82.2%)	0.307	0.003
COPD ***	54,792 (1.1%)	36,982 (19.8%)	0.643	0.198	54,792 (63.9%)	36,982 (77.8%)	0.310	0.006
Asthma	57,686 (1.1%)	49,596 (26.6%)	0.792	0.239	6886 (8.0%)	11,266 (23.7%)	0.439	0.004
Respiratory failure	11,499 (0.2%)	4562 (2.4%)	0.194	0.091	3324 (3.9%)	3866 (8.1%)	0.180	0.007
Lung cancer	10,323 (0.2%)	1743 (0.9%)	0.097	0.010	2383 (2.8%)	1314 (2.8%)	0.001	0.002
Rheumatologic or immunological disease	228,940 (4.5%)	15,100 (8.1%)	0.147	0.022	11,259 (13.1%)	5948 (12.5%)	0.018	0.006
CKD	60896 (1.2%)	4103 (2.2%)	0.077	0.001	4808 (5.6%)	2223 (4.7%)	0.042	0.001
Kidney failure	85,588 (1.7%)	6103 (3.3%)	0.102	0.008	7019 (8.2%)	3399 (7.1%)	0.039	0.003
**Prescribed medications**								
Oral corticosteroids	289,806 (5.7%)	54,109 (29.0%)	0.645	0.232	17,899 (20.9%)	19,724 (41.5%)	0.457	0.009
Biologics	618 (0.0%)	221 (0.1%)	0.042	0.024	11 (0.0%)	24 (0.1%)	0.021	0.006
Inhaled SABA	219279 (4.3%)	110,750 (59.4%)	1.463	0.299	26,885 (31.3%)	29,891 (62.9%)	0.666	0.002
Inhaled LABA	123119 (2.4%)	139,763 (74.9%)	2.228	0.294	27,281 (31.8%)	41,801 (87.9%)	1.395	0.001
Inhaled SAMA	6664 (0.1%)	7528 (4.0%)	0.276	0.162	2613 (3.0%)	4560 (9.6%)	0.271	0.001
Inhaled LAMA	66752 (1.3%)	50,416 (27.0%)	0.793	0.245	41,908 (48.8%)	35,551 (74.8%)	0.553	0.005
LTRA	15769 (0.3%)	24,334 (13.0%)	0.527	0.223	941 (1.1%)	3567 (7.5%)	0.32	0.010
Immunosuppressants	232,157 (4.6%)	36,636 (19.6%)	0.474	0.191	13,878 (16.2%)	14,267 (30.0%)	0.333	0.011
Statins	916,200 (18.1%)	51,190 (27.4%)	0.224	0.072	34,745 (40.5%)	18,493 (38.9%)	0.033	0.006
**Other characteristics**								
Body mass index			0.129	0.085			0.044	0.006
Underweight	16,098 (0.3%)	2405 (1.3%)			2888 (3.4%)	1837 (3.9%)		
Normal	179,854 (3.6%)	21,336 (11.4%)			17,831 (20.8%)	9970 (21.0%)		
Overweight	275,816 (5.5%)	27,131 (14.5%)			18,411 (21.5%)	9941 (20.9%)		
Obese	366,259 (7.2%)	32,455 (17.4%)			17,192 (20.0%)	10,100 (21.2%)		
Missing	4,218,875 (83.4%)	103,250 (55.3%)			29,491 (34.4%)	15,711 (33.0%)		
Smoking			0.357	0.142			0.207	0.014
Current	182,412 (3.6%)	17,169 (9.2%)			24,540 (28.6%)	10,274 (21.6%)		
Former	211,152 (4.2%)	30,148 (16.2%)			24,539 (28.6%)	15,991 (33.6%)		
Non-current	102,281 (2.0%)	3278 (1.8%)			1839 (2.1%)	994 (2.1%)		
Never	346,797 (6.9%)	33,874 (18.2%)			8774 (10.2%)	5533 (11.6%)		
Missing	4,214,260 (83.3%)	102,108 (54.7%)			26,121 (30.4%)	14,767 (31.0%)		
FEV_1_ predicted^a^							0.464	0.011
≥80	NA	NA			2443 (2.8%)	976 (2.1%)		
50–79	NA	NA			8247 (9.6%)	4325 (9.1%)		
30–49	NA	NA			2471 (2.9%)	2760 (5.8%)		
<30	NA	NA			290 (0.3%)	749 (1.6%)		
Missing	NA	NA			72,362 (84.3%)	38,749 (81.5%)		

**Notes**: *Non-exposed = received less than 3 prescriptions of ICS in the year before index (1 Jan 2020). **Exposed = received at least three filled ICS prescriptions within the year before the index (1 Jan 2020). ***Narrow COPD definition only by ICD-10 code J44. ^a^NA in the whole population as FEV_1_ was considered a covariate more relevant for COPD patients.

**Abbreviations**: COPD, chronic obstructive pulmonary disease; ICS, inhaled corticosteroids; ATT, average treatment for the treated; IHD, ischemic heart disease; CKD, chronic kidney disease; SABA, short-acting bronchodilator agonists; LABA, long-acting bronchodilator agonists; SAMA, short-acting muscarinic antagonists; LAMA, long-acting muscarinic antagonists; LTRA, leukotriene receptor antagonists; FEV_1_ predicted, post-bronchodilator Forced Expiratory Volume in one second, percent of predicted; SD, standard deviation.

### ICS-Exposure Related Risk for COVID-19 Outcomes in the Overall Cohort

The incidence rates of COVID-19 events were generally higher in the ICS-exposed compared to the non-exposed groups (Table S4). The ATT-weighting adjusted models showed weak and non-significant associations between previous ICS exposure and increased risk of COVID-19 onset, hospitalization, ICU admission and mortality in the whole population, and in COPD patients substantial associations similarly were not indicated for the studied outcomes, with a weak significant estimate only for hospitalization (Table 2).

**Table 2:** Hazard Ratios with 95 Confidence Intervals Comparing the Risk of Various COVID-19 Outcomes Between Individuals ≥40 Years of Age with Prior Regular Exposure to ICS to Non-Exposed Individuals, Adjusted for Multiple Confounders by Propensity Score Weighting ATT* Weighting

Study Cohorts	Study Population (Whole/COPD Subpopulation)	COVID-19 Outcome	HR [95 CI]	p value
Overall cohort	Whole population	Onset	1.02 [0.99–1.05]	0.158
		Hospitalization	1.05 [0.99–1.11]	0.087
		ICU admission	1.07 [0.89–1.29]	0.454
		COVID-19 Death	1.10 [0.99–1.22]	0.091
	COPD patients	Onset	1.07 [1–1.16]	0.061
		Hospitalization	1.13 [1.02–1.26]	0.025
		ICU admission	0.9 [0.61–1.32]	0.577
		Death	1.1 [0.93–1.3]	0.258
COVID-19 diagnosed sub cohort	Whole population	Hospitalization	1.04 [0.98–1.11]	0.148
		ICU admission	1.05 [0.86–1.27]	0.646
		Death	1.05 [0.93–1.18]	0.439
	COPD patients	Hospitalization	1.08 [0.98–1.19]	0.119
		ICU admission	0.83 [0.58–1.21]	0.332
		Death	0.94 [0.8–1.11]	0.474
COVID-19 hospitalized sub cohort	Whole population	ICU admission	0.98 [0.8–1.19]	0.816
		Death	0.97 [0.84–1.11]	0.639
	COPD patients	ICU admission	0.74 [0.51–1.08]	0.117
		Death	0.82 [0.67–0.99]	0.045

**Abbreviations**: *ATT, average treatment effect for the treated; COVID-19, Coronavirus disease, 2019; ICD-10, International Classification of Diseases version 10, Swedish Edition (ICD-10); HR, hazard ratio.

### ICS-Exposure Related Risk for COVID-19 Hospitalization or Death in Those with Diagnosed COVID-19 (COVID-19 Diagnosed Sub-Cohorts)

No significant associations between previous ICS exposure and hospitalization, admission or mortality were observed in the ATT-weighting adjusted models in COVID-19 patients from the whole population or from the COPD population in the COVID-19 diagnosed sub-cohort (Table 2).

### ICS- Exposure Related Risk for COVID-19 ICU Admission or Death in Hospitalized COVID-19 Patients (COVID-19 Hospitalized Sub-Cohorts)

**Figure 1:**
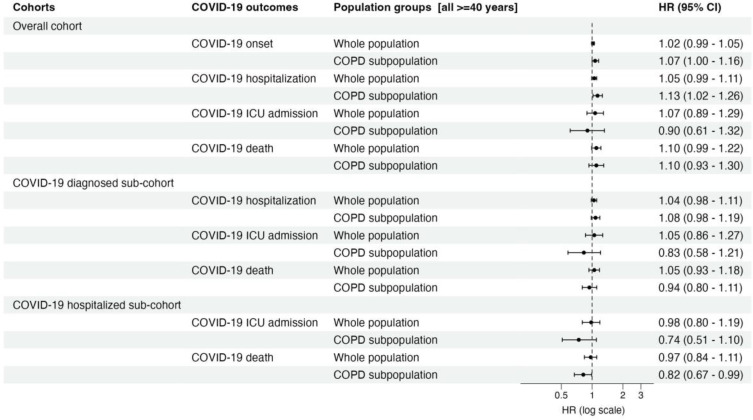
Forest plot comparing the risk of various definitions of COVID-19 between persons with pre-index exposure to ICS to non-exposed persons. The figure summarizes Average treatment for the treated (ATT) adjusted hazard ratios with 95% CI.

**Abbreviations**: HR, hazard ratio; CI, confidence interval; ICU, intensive care unit; COPD, chronic obstructive pulmonary disease; COVID-19, Coronavirus disease.

In the whole population hospitalized sub-cohort, no significant association was observed in the ATT adjusted models between ICS therapy and ICU mortality or death (Table 2). The point estimates were both close to 1 and non-significant. In contrast, the COVID-19 hospitalized sub-cohort among COPD patients showed reduced risk for ICU admission and mortality in patients receiving previous ICS therapy with significant protection for mortality (HR 0.82 95% CI [0.67–0.99]) (Table 2). A trend of shifting risk towards protection with more severe COVID-19 in COPD patients can thus be observed (Figure 1).

## Discussion

This study shows only weak non-significant associations between regular ICS exposure and COVID-19 onset, hospitalization, ICU admission and mortality in the overall cohort from the whole population and similarly in the overall COPD patient cohort, but not in the COPD patient population overall cohort or sub-cohorts in which regular ICS use appears even to be protective against severe COVID-19 disease, in particular against mortality in COPD patients hospitalized for COVID-19.

Ongoing ICS treatment may confer some risk of developing both mild and severe COVID-19, especially in a mixed whole population. In vitro studies have suggested that corticosteroids may impair antiviral innate immune response^21^,^22^ and that ICS use leads to delayed virus clearance.^23^ This could be a possible explanation to a slightly more frequent mild (onset) and more severe outcomes in the overall cohorts that we observed also in this study, although these effects were weak and non-significant. The observed associations could also partly be explained by underlying health differences between the exposed, who tended to have more comorbidities and higher medication use, and the non-exposed, despite extensive covariate balancing of the two groups by use of propensity score methods. Overall, however, we observed little evidence of COVID-19 risk from ICS use for COPD patients, especially in the diagnosed and hospitalized COPD sub-cohorts who might have a higher need of such treatment, and thus a better risk-benefit profile in terms of preventing or alleviating COVID-19-related exacerbations by consistent ICS use.

Indeed, ICS treatment might have benefit on cardiovascular related mortality in COPD as recently shown in large trials comparing triple inhaled combination therapy (including bronchodilators and ICS) with dual inhaled therapy (including bronchodilators only).^24^,^25^ This is relevant in the context of COPD, ICS and COVID-19, as cardiovascular disease is the most common comorbidity in patients with COPD and a risk factor for worse COVID-19 outcome. Other evidence has also suggested that ICS treatment in active COVID-19 disease or in susceptible populations may hinder progression to severe disease in the whole population. For example, Song et al reported that 14-day ciclesonide inhalation initiated after COVID-19 onset shortened SARS-CoV-2 viral shedding duration, and possibly inhibited the progression to acute respiratory failure in patients with mild-to-moderate COVID-19.^26^ Similarly, the STOIC trial found that administering inhaled budesonide to adult COVID-19 outpatients reduced the need for emergency visits and speeded up recovery, being an effective treatment of early COVID-19 and even affecting the rate of the persistent long-term symptoms in COVID-19.^27^ A study by Baker et al based on STOIC showed that enhanced immune response is activated already in early COVID-19 in the upper airway, and can be measured up to 35 days after the initial infection. According to the authors, it is possible to predict which patients will clinically deteriorate, by noting a blunted interferon and an exaggerated CCL24 airway inflammatory response. However, the inflammatory pathways and patterns of inflammation in the upper respiratory tract and circulation following COVID-19 infection were modulated by inhaled budesonide.^28^ Clemency et al showed that participants who were treated with ciclesonide had fewer subsequent emergency department visits or hospital admissions for reasons related to COVID-19 (odds ratio, 0.18; 95% CI, 0.04–0.85).^29^ One of the explanations might be that ICS modulate the inflammatory response. Taking ICS may be beneficial in dealing with virus infections, specifically coronavirus. As mentioned, ciclesonide blocks SARS-CoV-2 RNA replication in vitro^9^ and inhibits SARS-CoV-2 cytopathic activity,^30^ reducing thus the risk of developing severe COVID-19. Previously, both the PRINCIPAL and COVERAGE trials suggested that it was difficult to show that early administration of inhaled corticosteroid reduce the risk of clinical worsening in high-risk COVID-19 positive populations which includes COPD patients.^31^,^32^ In fact, our results show that among COPD patients hospitalized for COVID-19 there was even a clear protective effect. This may be related to the consistent ICS intake during the COVID-19 pandemic, since the prior treatment is most often continued and likely provide protection during this time frame. Part of this protective effect may also be explained by the fact that ICS therapy downregulates the SARS-CoV-2 receptors ACE2 and ADAM17,^33^ for which inhibition seems to be related to COVID-19 susceptibility and severity.^34^ Therefore, the ICS-mediated downregulation of ACE2 is thought to be protective, and COPD patients should definitely continue ICS both in general (prior to COVID-19) and after having developed COVID-19.

Regarding severe clinical outcomes or mortality, inconsistent results have been reported. Sen et al showed that prior ICS therapy did not increase COVID-19 related healthcare utilization or mortality outcome in patients with COPD.^35^ Bloom et al similarly showed neither benefit nor harm from ICS in patients with both COPD and asthma who were hospitalized with COVID-19 when it comes to inpatient clinical outcomes.^36^ Schultze et al, on the other hand, reported increased risk of mortality among patients with COPD on ICS although sensitivity analyses suggested this was from unmeasured confounding due to reduced baseline health status in patients on ICS.^11^ Aveyard et al studied COPD patients hospitalized with COVID-19 where prior ICS therapy was associated with a modest risk of severe COVID-19. The estimated risk was reduced but not normalized when adjusted for comorbidities and demographic factors.^37^ Important factors related to ICS use in relation to COVID-19 are the timing of ICS use, as well as the underlying indication for usage, which may explain the heterogenous results. Regular prior use (for an underlying condition) has been shown to have a different effect than administration at or after time of infection.^38^ Our results are consistent with this view, and suggest that conditional on contracting COVID-19, a prior treatment with ICS is protective – possibly related to continued administration during the COVID-19 infection.

This study has several strengths, including a large sample size, high data completeness for most covariates except for some patient factors with some missing data, and the capability of assessing whether prior use of ICS was associated with outcomes in a nationwide cohort reflecting both the whole population and COPD patients with COVID-19. Another important strength of our study is the use of nationwide register data, which minimized the possibility of selection bias. However, this study also has limitations. Inherent to its retrospective observational design, causal interpretation of the findings is still subject to residual bias from potential residual or unmeasured confounding. For example, patients with lung diseases or ICS treatment may have other comorbidities or characteristics that are not captured in the data that may bias the risk estimates. Nevertheless, by our propensity score analysis, we adjusted for a wide range of comorbidities and markers of disease severity and treatment and achieved quite good balance between compared groups in all analyses, so that major residual confounding is unlikely. We also lacked some important data on COPD severity for all COPD individuals that would have been desirable for our analyses, eg, Global Initiative for Chronic Obstructive Lung Disease (GOLD) grouping, but available FEV_1_ data were a good surrogate despite some missingness. Another limitation is the considerable amount of missing data on key variables like smoking, BMI and FEV_1_. Although we used analytical methods to deal with missing values, it might impact on the validity of the results. Similarly, although register data captures data on prescription drugs very well, some ICS are also available over the counter and may not have been captured in our data.

## Conclusion

In summary, this large population study suggests that the benefits of ongoing ICS therapy in COPD patients outweighs the potential associated risks. There is no evidence to support discontinuation of ICS among patients with COPD for COVID-19-related reasons. On the contrary, our results provide support for the continuation of ICS treatment for COPD patients, considering the protective effect in reducing severe disease, including mortality.
